# Seasonal Breeding Alters Fecal Microbiota and Metabolome in the Male Captive Yangtze Finless Porpoise (*Neophocaena asiaeorientalis asiaeorientalis*)

**DOI:** 10.1002/ece3.71611

**Published:** 2025-06-22

**Authors:** Syed Ata Ur Rahman Shah, Bin Tang, Dekui He, Maaz Ahmad, Ghulam Nabi, Chaoqun Wang, Zhangbing Kou, Kexiong Wang, Yujiang Hao

**Affiliations:** ^1^ Key Laboratory of Aquatic Biodiversity and Conservation, Institute of Hydrobiology Chinese Academy of Sciences Wuhan China; ^2^ University of Chinese Academy of Sciences Beijing China; ^3^ National Aquatic Biological Resource Center (NABRC) Wuhan China; ^4^ Department of Zoology, Institute of Molecular Biology and Biotechnology University of Lahore Lahore Pakistan

**Keywords:** fecal metabolome, fecal microbiome, seasonal breeding, Yangtze finless porpoise

## Abstract

The Yangtze finless porpoise (YFP) is a critically endangered freshwater cetacean endemic to China. Understanding seasonal breeding patterns is critical for the effective conservation of critically endangered species. The current study was designed to examine the function and taxonomic characteristics of fecal microbiota and their metabolites in male captive YFPs during both nonbreeding (NB) and breeding (B) seasons, analyzing 20 fecal samples using both UHPLC–MS/MS and 16S rRNA gene sequencing approaches. The present study revealed that Firmicutes were increased in the NB season, while Actinobacteria, Proteobacteria, and Fusobacteriota were increased in the B season at the phylum level. At the genus level, *Paeniclostridium*, *Clostridium_sensu_stricto_13*, and *Mycobacterium* were increased in the NB season, while *Romboutsia*, *Plesiomonas*, and *Cetobacterium* were increased in the B season. LEfSe analysis revealed that *Staphylococcus*, *Comamonas*, and *Tetrasphaera* were significantly increased in the B season, while the genus *Terrisporobacter* was substantially increased in the NB season. The fecal metabolome undergoes significant changes during the B and NB seasons, altering metabolic pathways such as phenylalanine metabolism, protein digestion, taurine and hypotaurine metabolism, lysine degradation, tryptophan biosynthesis, tyrosine metabolism, and bile secretion. Moreover, there was a significant correlation between the fecal metabolome and microbiome in the captive YFPs in the B and NB seasons. This study explores the impact of seasonal reproduction on gut microbes and their metabolites, providing insights into animal seasonal reproductive behavior and providing a theoretical basis for studying gut microbiota and metabolites in cetaceans, both in captivity and in the wild.

## Graphical abstract

This study investigates the seasonal variations in fecal microbiota and metabolites of captive Yangtze finless porpoises (YFP) during breeding (B) and nonbreeding (NB) seasons using 16S rRNA gene sequencing and UHPLC–MS/MS analysis. Firmicutes were dominant in NB, while Actinobacteria, Proteobacteria, and Fusobacteriota increased in B. Significant shifts in microbial composition and metabolic pathways, including phenylalanine metabolism and bile secretion, were observed. Correlations between microbiota and metabolites highlight the influence of seasonal reproduction on gut ecology, providing insights into cetacean reproductive biology in captivity and the wild.

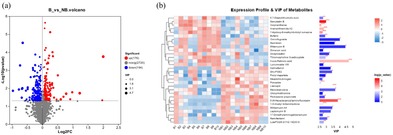

## 1 Introduction

The gut microbiota, a distinct group of microorganisms in animals' digestive systems, is crucial for their metabolism, reproduction, and immunity (Hsiao et al. 2008; Ley et al. 2006; Morrison and Preston 2016). The gut microbiota is crucial for energy supply, secreting up to 35% of metabolic and digestive enzymes in mammals (Gill et al. 1979). It is diverse, unstable, and susceptible to external influences like food, age, disease, and habitats (Fan et al. 2022; Guo et al. 2021; O'Toole 2012; Gomaa 2020; Sun et al. 2020; Wong and Rawls 2012), influencing host energy metabolism and adaptation. Currently, some studies have found that gut microbiota is strongly linked to animal reproduction, as it plays a role in the deglucuronidation of testosterone and dihydrotestosterone (DHT) and metabolism. Young, healthy mice have higher DHT levels in their colon than germ‐free mice (Colldén et al. 2019), suggesting that further research on gut microbiota composition and function is important for the reproduction of animals.

Seasonal breeding is a natural process where certain animals breed at particular periods of the year, caused by photoperiodism (Matthews et al. 1993). Seasonal reproductive variations are caused by the pineal gland in the brain, which detects these changes and produces melatonin at night to control the release of luteinizing and gonadotropin‐releasing hormones (Malpaux et al. 2002). Seasonal breeding in animals is influenced by a variety of variables, including living conditions, thyroid hormone, estrogen, and kisspeptin (Dardente et al. 2016; Nakao et al. 2008; Smith 2012; Smith and Clarke 2010). Gut microbiota interacts with estrogens through the estrobolome gene, which can metabolize estrogen (Baker et al. 2017; Plottel and Blaser 2011). The secretion of β‐glucuronidase by gut microbiota affects estrogen levels by binding to estrogen receptor alpha and beta, affecting downstream physiological effects (Baker et al. 2017). Recent research suggests that gut microbiota may control behavior and seasonal breeding via the melatonin, hypothalamic–pituitary–gonadal (HPG) axis, and the kisspeptin/G‐protein‐coupled receptor 54 system in the hypothalamus of rodents (Zhu et al. 2022). This generates curiosity in learning more about the relationship between seasonal breeding and fecal microbiota and its metabolites in the male captive YFPs.

The Yangtze finless porpoise, the sole freshwater porpoise in the world, has been classified as “Endangered” on the IUCN Red List since 2017 with a decreasing population (https://doi.org/10.2305/IUCN.UK.2017‐3.RLTS.T41754A50381766.en, accessed on May 02, 2025) (Wang and Reeves 2017). Since the extinction of baiji in 2006, this species became the last remaining cetacean in the Yangtze River, now limited to the main river section downstream of the Gezhouba Dam and its adjacent lakes (Turvey et al. 2007; Wang 2009). The YFP is a seasonal breeder, with an NB season from November to February and a B season from March to September (Hao et al. 2007; Daoquan et al. 2006). Seasonal reproduction in cetacean species is typically assessed through endocrinological monitoring (Robeck et al. 2001; Atkinson and Yoshioka 2007; Hao et al. 2019). Daoquan et al. (2006) and Hao et al. (2019) confirmed reproductive seasonality in mature male YFPs, with increased serum testosterone concentrations in males from March, dropping in September, and remaining quiescent in the rest of the months. However, it is still unknown how fecal microbiota and its metabolites influence seasonal breeding in cetaceans and specifically in the captive YFP. So, this is the first‐ever study aimed at exploring the influence and strong association of fecal microbiota and metabolome in the captive YFPs during distinct breeding seasons. The present study provides a new theoretical basis for researchers studying captive YFP or other cetaceans’ reproductive behavior, and fecal microbiota and its metabolites can be used to explain the seasonal breeding mechanisms.

## 2 Materials and Methodology

### 2.1 Collection of Fecal Samples

Two male dolphins, TT and DD, were used to collect fecal samples. TT was born in 2005 at the Baiji Dolphinarium, Wuhan, China, and DD was transported from Poyang Lake in 2011. We collected 20 fresh fecal samples from both animals during the B and NB seasons, between June 2021 and May 2022 for TT and June 2022 to May 2023 for DD, as shown in the (Table S1). The animals were living in the same big kidney‐shaped pool (20 × 7 × 3.5 m), with water recycled 10 times a day. During the breeding season, males and females cohabitated to facilitate successful mating, while in nonbreeding seasons, the males were separated from females to accommodate pregnant animals. The animals had direct physical contact when housed together. The animals were given a daily intake of several fish species, such as crucian carp ( *Carassius auratus* ), common carp ( *Cyprinus carpio* ), and sharp belly ( *Hemiculter leucisculus* ), which accounted for 6%–8% of their total weight. During the sample procedure, neither animal received any antibiotics or drugs, and both animals were in good health. The temperature ranged from 7°C to 27°C. Fecal samples were collected from the animals using operant training (Hao et al. 2019). In order to get fecal samples, a trainer softly tickled the animal's anal area and stimulated it to turn around its belly. Samples were frozen and kept for further examination at −80°C after the animal ID and date were recorded. The methodology was authorized by the Ethics Committee Research of the Chinese Academy of Sciences and the Institute of Hydrobiology, Wuhan, China (IHB/LL/2022‐YFF1301604), ensuring adherence to wildlife ethics and Chinese laws.

### 2.2 DNA Extraction and PCR Amplification

Every fecal sample's bacterial genomic DNA was extracted using the Quick‐DNA TM Fecal/Soil Microbe DNA MiniPrep Kit (Zymo Research) in accordance with the company's instructions. The universal bacterial primer sets 338F and 806R were used for bacterial 16S rRNA gene amplification in the V3–V4 region. A prior work by Wan et al. (2022) describes the procedures for PCR amplification and purification. A NanoDrop 2000 (Thermo Scientific, Wilmington, USA) was used to evaluate the yield and purity of the DNA.

### 2.3 Sequence Processing and Bioinformatic Analysis

The purified amplicons were adjusted to an equivalent concentration and then sequenced using the Illumina NovaSeq6000 system (Illumina, San Diego, CA, USA), as was previously mentioned (Shah et al. 2025). Using default parameters, the raw sequencing data were processed by DAD2 in QIIME2 (v2019.10) (Bolyen et al. 2019; Callens et al. 2016). In line with earlier research (Shah, Tang, He, Hao, Ahmad, et al. 2024; Shah, Tang, He, Hao, Nabi, et al. 2024), all sample reads were then annotated and aligned with the Silva database (v138.1, 16S rDNA) (Quast et al. 2012). Principal coordinate analysis (PCoA) based on Bray–Curtis dissimilarity was used to analyze the microbial communities in all the samples using the Vegan (v2.5‐3) program. The alpha diversity index, which is based on the OTUs information and includes Shannon, Simpson, ACE, Chao1, and good coverage, is constructed using Mothur (v1.30.1). Significant bacterial species were identified in both seasonal groups using the linear discriminant analysis (LEfSe) effect size (*p* < 0.05 and LDA score > 2.0) (Díaz‐Sánchez et al. 2019). The R program ggplot 2 was utilized for plotting, and the Benjamini–Hochberg (BH) method was utilized for adjusting *p*‐values (Benjamini and Hochberg 2000).

### 2.4 Analysis of Untargeted Metabolomics

UHPLC–MS/MS nontargeted metabolomics were used to identify fecal metabolites. A 100 μL fecal sample was weighed and extracted using a 4:1 v/v methanol/water solution. A high‐throughput tissue crusher (Wonbio‐96c, Shanghai Wanbo Biotechnology Co. Ltd.) was utilized to vortex the mixture for 30 s, treat it for 6 min at 50 Hz, and then subject it to ultrasonic treatment for 30 min at 40 kHz and 5°C. At −20°C, protein precipitation was done for 30 min. Before being put into sample vials for LC–MS/MS analysis, the supernatant was centrifuged at 13,000 g for 15 min at 4°C. The raw data entered into Progenesis Q1 2.3 were utilized to conduct the LC–MS/MS analysis. The peak intensity, retention period, and mass‐to‐charge ratio were all included in the final data matrix. The metabolic traits of every sample group were preserved to a minimum of 80%. By normalizing each metabolite characteristic using summing after filtering, the lowest metabolite value for samples below the quantitative lower limit was found. Metabolite characteristics having a relative standard deviation (RSD) of QC > 30% were excluded from the data quality evaluation based on internal standards. The Human Metabolome Database (https://www.hmbd.ca/) and Metlin (https://metlin.scripps.edu/) are reliable biochemical databases that were utilized to find MS/MS fragment spectra, precise mass, and isotope ratio changes. The R package ropls (Version 1.6.2) and the Majorbio Cloud platform (https://cloud.majorbio.com) were used to perform the multivariate statistical analysis.

### 2.5 Data Processing and Statistical Methods

Potential relationships between fecal bacteria and metabolites were analyzed using Spearman rank correlation in the R psych package (Revelle 2020). Only bacterial–metabolite pairs with significant correlations (*p*‐value < 0.05) were retained for further analysis.

## 3 Results

The present study generated 1,473,092 raw 16S rRNA reads (mean: 73,654.6; range: 56,731–191,108) from 20 fecal samples, categorized into 22 phyla, 58 classes, 150 orders, 257 families, 439 genera, 639 species, and 1000 OTUs. All samples showed a saturation plateau in the Shannon rarefaction curve (Figure S1a). Firmicutes, Proteobacteria, Actinobacteriota, and unclassified_k_norank_d_Bacteria were the top phyla, while *Paeniclostridium*, *Clostridium_sensu_stricto_13*, *Romboutsia*, *Clostridium_sensu_stricto_1*, and *Ureaplasma* were the highest genera. In the B and NB seasons, the Venn diagram identified 456 common OTUs and 290 and 254 distinct OTUs, respectively (Figure S1b).

### 3.1 Alpha and Beta Diversity of the Gut Microbiota in the Two Seasonal Groups

**FIGURE 1:**
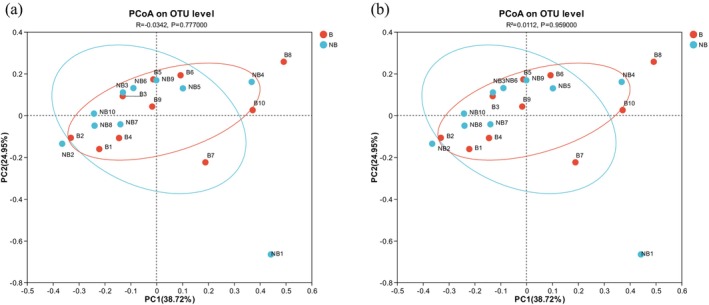
The PCoA utilizing Bray–Curtis distance (a) ANOSIM, (b) ADONIS, illustrates changes in the fecal bacterial community composition of captive YFP during two breeding seasons.

The current research reported that the Simpson diversity index was larger in the NB season, while the ACE richness estimator, Shannon index, Chao index, and sobs were increased in B season (Table S2 and Figure S2a–d). However, there was no discernible change in community diversity as indicated by the lack of substantial variations in the Chao, Simpson, and Shannon indices. The current study analyzed fecal microbiota composition differences among captive YFPs using the Bray–Curtis (anosim and ADONIS) methodologies. It found no significant (*p* > 0.05) change in the composition of gut microbiota between the B and NB seasonal groups (Figure 1a,b).

### 3.2 Composition of Gut Microbiota Species and Comparison of the Two Breeding Seasons

**FIGURE 2:**
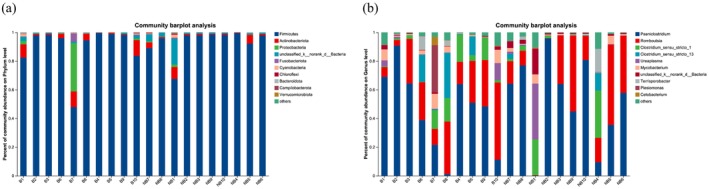
The current research showed variation in the richness of fecal microbiota between two breeding seasons, with a histogram displaying the relative richness of the top 10 bacterial (a) phyla and (b) genera.

Firmicutes (94% and 90%), Proteobacteria (0% and 4%), Actinobacteria (2% and 4%), unclassified_k_norank_d_Bacteria (3% and 1%), and Fusobacteriota (0% and 1%) were the primary groups identified at the phylum level in the two breeding seasons' captive YFP fecal microbiota analysis (Figure 2a). The abundant bacterial genera were *Paeniclostridium* (53% and 46%), *Romboutsia* (24% and 25%), *Clostridium_sensu_stricto_13* (7% and 2%), *Clostridium_sensu_stricto_1* (7% and 7%), *Ureaplasma* (4% and 2%), *Mycobacterium* (4% and 2%), *norank_d__Bacteria* (3% and 1%), *Plesiomonas* (0% and 3%), *Terrisporobacter* (2% and 1%), *Clostridiaceae* (1% and 0%), *Cetobacterium* (0% and 1%), and *Epulopiscium* (1% and 0%) in the NB and B groups, respectively (Figure 2b).

### 3.3 Differential Bacterial Biomarkers in the Two Seasonal Groups

**FIGURE 3:**
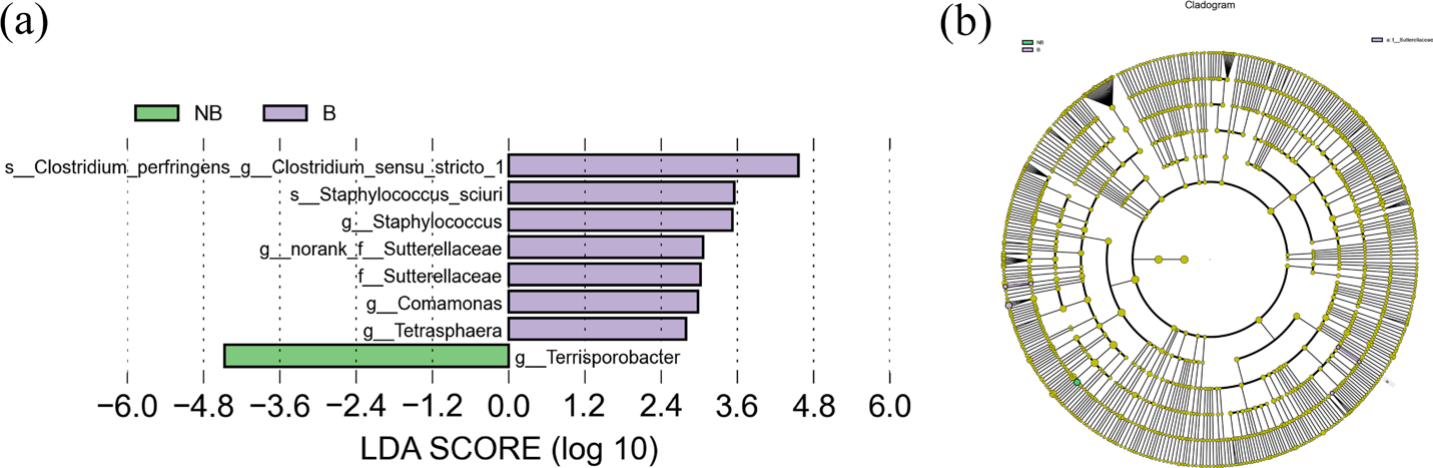
The fecal microbiota of the two breeding seasons in the captive YFP contain different genera of bacteria. (a) A significant alteration in abundance between B and NB seasons bacteria is revealed by LEfSe analysis (LDA > 2.0). (b) The cladogram of bacterial species reveals notable variations in abundances between the two breeding seasons.

The study utilized LEfSe analysis to find microorganisms with substantial differences in relative richness across two breeding seasons (LDA > 2 and *p* < 0.05). The study reveals that *Staphylococcus*, *Sutterellaceae_unclassified*, *Comamonas*, and *Tetrasphaera* increased significantly in the B season, while *Terrisporobacter* increased significantly in the NB season (Figure 3).

### 3.4 Fecal Metabolome in the Two Breeding Seasons

**FIGURE 4:**
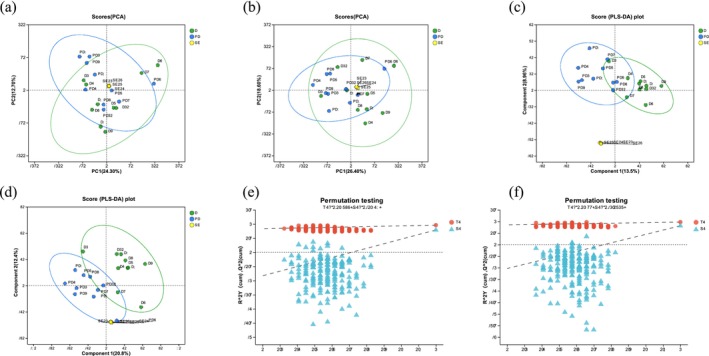
Processing high‐quality metabolomics data. Principal component analysis (PCA) scores were generated for samples collected in (a) positive ionization mode and (b) negative ionization mode. The confidence ellipse indicates a 95% confidence level for the distribution of “real” samples within the designated area. The more distinct the two sample groups are from one another, the more significant the classification impact is. The PLS‐DA scores are presented for (c) positive ions and (d) negative ions. The permutation test uses the abscissa to represent the reservation, while the regression lines for *R* ^2^ (the red dot) and *Q* ^2^ (the blue triangle) are shown on the ordinate, representing the PLS‐DA permutation test in the (e) positive and (f) negative ion modes.

One thousand seven hundred twenty metabolites were found in the positive ionization mode and 1375 metabolites in the negative ionization mode in the current study (Table S3). QC samples showed strong clustering near the plot's origin, indicating great stability and repeatability. The two seasonal breeding groups' metabolites were identified using the supervised PCA model (Figure 4a,b), whereas the PLS‐DA findings revealed different metabolites (Figure 4c,d). The PLS‐DA model findings showed that the model was suitable for additional data analysis, fit well, and was very predictable (Figure 4e,f). The intercepts for the vertical axis *Y* and the regression line of *Q* ^2^ are 0.8364 and 0.855, respectively. *R* ^2^ values are higher than *Q* ^2^ for both the positive and the negative ion models.

### 3.5 Differential Metabolites in the Two Breeding Seasons

**FIGURE 5:**
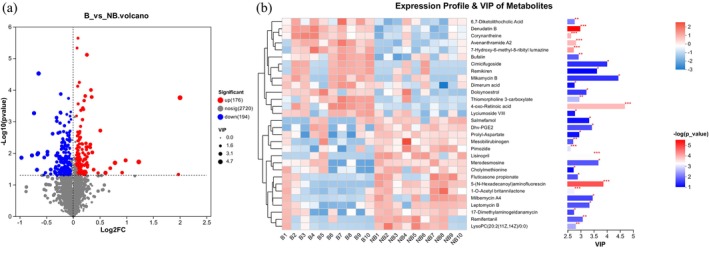
Examining the two breeding seasons for significant differences in metabolites. (a) The volcano represents the distinct metabolites for the B and NB seasonal groups. The *y*‐axis represents the statistical significance of differential metabolite expression, expressed as ‐log10 (*p*‐value), while the *x*‐axis, or log2FC (log2 fold change), illustrates the magnitude of change in metabolite expression between the two breeding seasons. Higher values indicate greater significance in expression differences. (b) DMs of captive YFP in the B and NB groups were identified through cluster analysis. The left side displays a clustering dendrogram of the metabolites, where the proximity of branches reflects the similarity in expression patterns among metabolites within the samples. On the right, a VIP (variable importance in projection) bar chart is shown, with bar length representing the contribution of each metabolite to the differences between the two groups. By default, no values are below 1, and higher values indicate greater disparities between the groups. The color intensity of the bars corresponds to the significance of the differences, with darker colors and lower *p*‐values indicating higher −log10 values. Significance levels are denoted by *, **, and *** for *p* < 0.05, *p* < 0.01, and *p* < 0.001, respectively.

The two seasonal breeding groups' differential metabolites (DMs) were identified using the OPLS‐DA model based on a *p*‐value < 0.05 and VIP > 1 (Figure 5). Three hundred seventy DMs were found in the NB and B groups (Table S4). Among them, 176 DMs, that is, 6,7‐diketolithocholic acid, denudatin b, corynantheine, avenanthramide a2, 7‐hydroxy‐6‐methyl‐8‐ribityl lumazine, bufalin, cimicifugoside, remikiren, mikamycin b, dimerum acid, doisynoestrol, thiomorpholine 3‐carboxylate, lyciumoside VIII, and 4‐oxo‐retinoic acid, were upregulated in the B group, while 194 DMs, that is, salmefamol, dhv‐PGE2, prolyl‐aspartate, mesobilirubinogen, pimozide, lisinopril, merodesmosine, 5‐(n‐hexadecanoyl)aminofluorescin, cholylmethionine, fluticasone propionate, 1‐o‐acetyl britannilactone, milbemycin a4, leptomycin b, 17‐dimethylaminogeldanamycin, lysoPC(20:2(11Z,14Z)/0:0), and remifentanil, were downregulated in the NB season.

### 3.6 Enrichment Analysis of Metabolic Pathways in the Two Breeding Seasons

**FIGURE 6:**
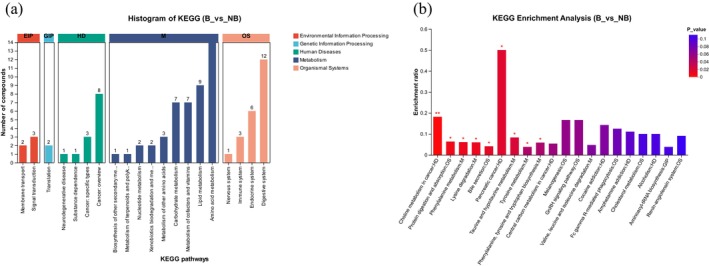
(a) Classification using the KEGG pathway: Metabolites identified and labeled in the captive YFP's two breeding seasons. The *x*‐axis shows the total number of metabolites found, and the *y*‐axis shows the KEGG pathway's level‐2 terms. (b) Enrichment of metabolic pathways of distinct metabolites between the B and NB seasons in the captive YFP. The symbols * and ** indicate a significance level of *p* < 0.05, and *p* < 0.01, respectively.

The present research found metabolites in second‐grade KEGG pathways, with amino acid, cofactor and vitamin, lipid, carbohydrate, and nucleotide metabolism being the most substantial (Figure 6a). The enrichment analysis reported substantial alterations between B and NB seasonal groups in metabolic pathways, including protein digestion and absorption, choline metabolism in cancer, phenylalanine metabolism, lysine degradation, tyrosine metabolism, taurine and hypotaurine metabolism, bile secretion, pancreatic cancer, and phenylalanine, tyrosine, and tryptophan biosynthesis (Figure 6b and Table S5).

### 3.7 Fecal Microbiome and Metabolome Correlation in the Two Breeding Seasons

**FIGURE 7:**
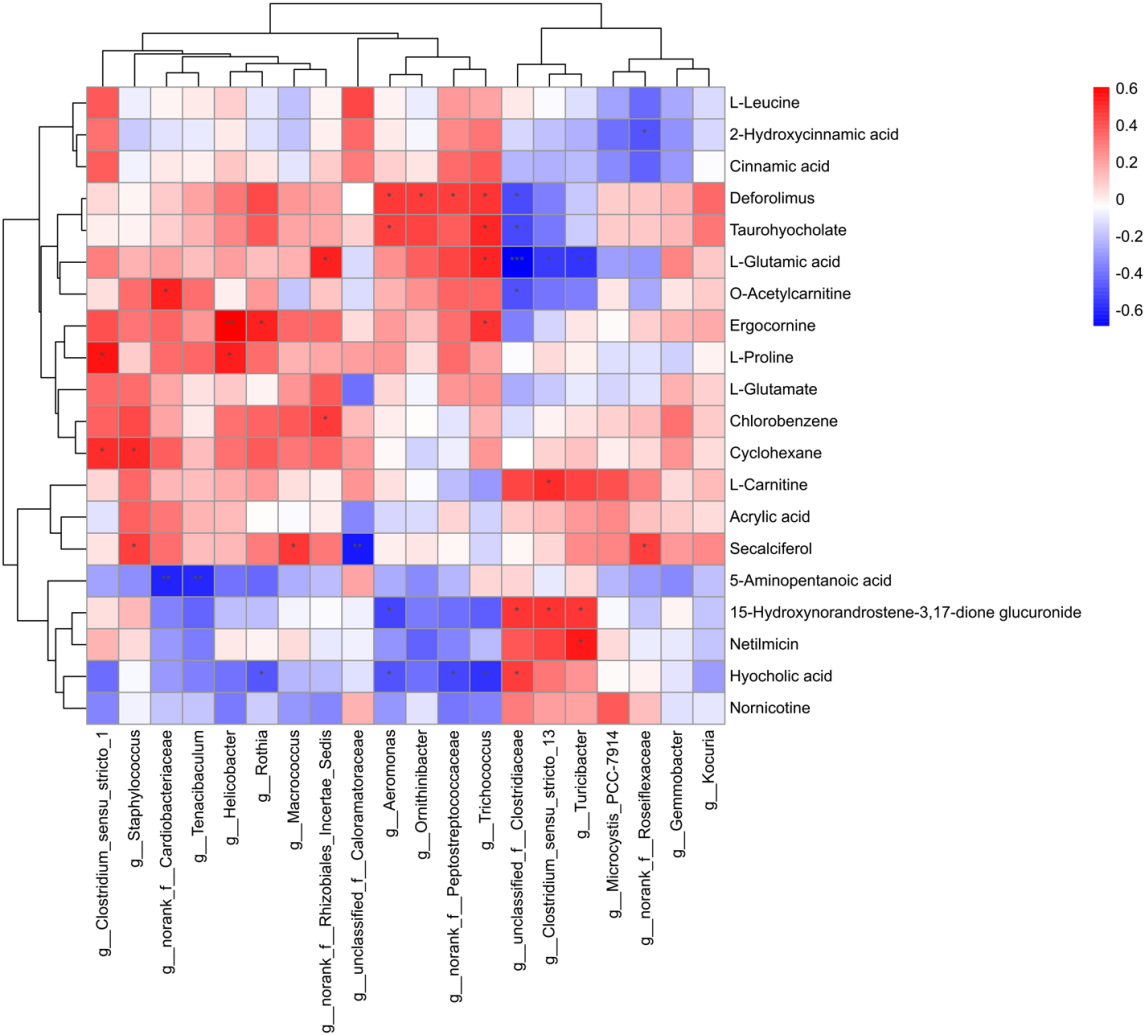
Multiomics methods were used to identify microbiota–metabolite interactions in the two breeding seasons of the captive YFP. Heatmap illustrating the association between fecal microbiota and metabolite clusters. Different colors indicate the size of the Spearman correlation coefficient. Blue denotes a negative correlation, whereas red denotes a positive one. The asterisks indicate statistically significant correlations (*p* < 0.05) between metabolite clusters and bacteria, whereas the symbols *, **, and *** indicate significance levels of *p* < 0.05, *p* < 0.01, and *p* < 0.001, respectively.

The present study reported a significant correlation among the top 20 metabolites and microbes by utilizing Spearman correlation analysis (Figure 7). The present study found that *Clostridium_sensu_stricto_1* was substantially positively correlated with l‐proline and cyclohexane, while *Staphylococcus* and *Macrococcus* were positively correlated with secalciferol. *Cardiobacteriaceae* was positively correlated with o‐acetylcarnitine, *Helicobacter* was positively correlated with ergocornine and l‐proline, and *Rhizobiales_Incertae_Sedis* was positively correlated with l‐glutamic acid and chlorobenzene. *Aeromonas*, *Ornithinibacter*, *norank_f__Peptostreptococcaceae*, and *Trichococcus* were positively correlated with deforolimus, taurohyocholate, l‐glutamic acid, and ergocornine. *Clostridium_sensu_stricto_13* and *Turicibacter* were positively correlated with l‐carnitine and 15‐hydrooxynorandrostene‐3,17‐dione glucuronide, and netilmicin while negatively correlated with l‐glutamic acid. *Roseiflexaceae* was positively associated with secalciferol, while negatively associated with 2‐hydroxycinnamic acid. *Cardiobacteriaceae* and *Tenacibaculum* were negatively associated with 5‐aminopentanoic acid, while *Rothia*, *Aeromonas*, *norank_f__Peptostreptococcaceae*, and *Trichococcus* were negatively associated with hyocholic acid.

## 4 Discussion

Seasonal breeding is a natural process where animals adapt to natural selection and reproduce exclusively during specific seasons. The ability of individuals to adjust to seasonal fluctuations and variations in the distribution, availability, and amount of food is essential for their survival and reproduction (Sun et al. 2016). The YFP is a seasonal breeder, with an NB season from November to February and a B season from March to September (Hao et al. 2007; Daoquan et al. 2006). Numerous studies have explored the seasonal changes in gut microbiota in muskrats ( *Ondatra zibethicus* ) (Song, Xu, et al. 2023) and wild ground squirrels ( *Spermophilus dauricus* ) (Yang et al. 2021; Song, Ma, et al. 2023). This is the first‐ever study that utilizes next‐generation sequencing technology and UHPLC–MS/MS approaches to analyze the fecal microbiota composition and metabolites in male captive YFP during B and NB seasons. The study found no substantial alterations in fecal microbiota structure and function, but the abundance varied between B and NB male captive YFP. The study suggests that fecal microbes may play a role in meeting the diverse needs of seasonal breeding in captive YFP.

The study found that the fecal microbiota structure of captive YFP may be influenced by seasonal variations. Alpha and beta diversities are crucial metrics for describing community diversity, focusing on species richness and evenness within a community. The alpha diversity study revealed higher gut microbial richness during B season compared to NB season, but no significant difference between seasons. As a result, we hypothesize that higher fecal microbiota abundance would help meet YFP's energy needs during mating. Studies have shown that alpha diversity in gut microbiota in Siberian chipmunks (Zhou et al. 2021) increases during hibernation, while alpha diversity changes in muskrats during nonbreeding seasons (Song, Xu, et al. 2023). The current research found that the alpha diversity of captive YFP fecal microbiota did not significantly change with seasonal breeding, indicating that the changes in breeding status did not impact the overall richness of their gut microbiota, consistent with previous studies by Song, Ma, et al. (2023) and Yang et al. (2021). The lack of significant seasonal variation may reflect unique adaptations of YFP's fecal microbiota, such as metabolic flexibility or dietary consistency in captivity, or it could stem from methodological factors like sample size limitations or individual variability. The present study found no substantial differences in beta diversity. This contrasts with previous studies revealing significant changes in beta diversity in muskrats ( *Ondatra zibethicus* ) (Song, Xu, et al. 2023) and wild ground squirrels ( *Spermophilus dauricus* ) (Yang et al. 2021; Song, Ma, et al. 2023). This difference may be due to the collection of fecal samples throughout the B and NB seasons, rather than a single timepoint.

Consistent with our earlier research, Firmicutes dominated the fecal content samples of both B and NB seasons of the captive YFP at the phylum level (Shah et al. 2025; Shah, Tang, He, Hao, Ahmad, et al. 2024; Shah, Tang, He, Hao, Nabi, et al. 2024). Indeed, Firmicutes are the main phylum in the gut of many other wild animals, including the snub‐nosed monkey (*Rhinopithecus* spp.) (Li et al. 2023), the Indo‐Pacific humpback dolphin ( *Sousa chinensis* ) (Wan et al. 2021), and other mammals (Ley et al. 2008). Firmicutes are microbes that produce short‐chain fatty acids (SCFAs) and break down carbohydrates in the gut to give energy to the host (Macfarlane and Macfarlane 2003). They have better efficiency in breaking down lipids and carbohydrates, making them more conducive to nutrient absorption (Magne et al. 2020). Firmicutes proliferate in rodents' guts as a result of a high energy expenditure (Lee et al. 2020), demonstrating that increased gut Firmicutes facilitate energy uptake. Firmicutes were abundant in the NB season compared to the B season, aligning with previous studies on goat semen (Mocé et al. 2022). Higher levels of Firmicutes in the NB season of male captive YFP may be due to their good appetite and energy storage needs during periods of lower reproductive activity, while also potentially influencing hormonal regulation and nutrient partitioning. Actinobacteria, a saccharolytic bacteria, produce SCFAs, which increase energy harvesting from diet and interfere with host energy homeostasis (Indiani et al. 2018; Murugesan et al. 2018). Numerous research studies have shown that Proteobacteria are linked to energy accumulation (Koren et al. 2012; Collado et al. 2008). The increased abundance of these species is beneficial as it helps the captive YFPs meet the energy demands of reproduction, such as spermatogenesis and mate competition, during B season. Proteobacteria are diverse species with varying metabolic capabilities for breaking down organic substances (Shin et al. 2015). The B season sees a higher abundance of Fusobacteriota, linked to high‐protein food consumption. Cetobacterium, a member of Fusobacteriota, is thought to play a role in lipid metabolism (Yao et al. 2023; Zhu et al. 2021). Actinobacteria, Proteobacteria, and Fusobacteriota were reported to be abundant in the B season in the semen of the goats (Mocé et al. 2022). These results suggest that these phyla may be associated with high energy supply during the B season of male captive YFP.

At the genus level, the NB season showed higher levels of *Paeniclostridium*, a pathogenic bacterium that induces inflammation in the intestinal tract and various animal tissues through the production of cytotoxic endotoxins (DeCandia et al. 2024; Nyaoke et al. 2020; Gryaznova et al. 2021; Watts et al. 2019; Li et al. 2022). *Romboutsia*, a genus associated with healthy hosts and a biomarker of intestinal dysbiosis, has a range of metabolic capabilities, including carbohydrate utilization, anaerobic respiration, single amino acid fermentation, and metabolic end‐products. It can generate SCFAs through dietary fermentation, providing energy and maintaining intestinal environment stability (Mangifesta et al. 2018; Gerritsen et al. 2019; Wang and Jia 2016). *Romboutsia* was increased in the B season as compared to the NB season in the captive YFP. *Clostridium_sensu_stricto_13*, a producer of SCFA, is found to be higher in the NB and has been identified as a beneficial bacterium for protecting the intestinal barrier (Kong et al. 2019). This aligns with recent findings reporting *Clostridiaceae*'s central role in aquatic mammals, including its dominance in rehabilitated Mediterranean monk seal pups (Dosi et al. 2024). Numerous studies found a link between *Ureaplasma* and male infertility in humans (Gdoura et al. 2007; Huang et al. 2015) which was abundant in the NB season as compared to the B season. During the B season, the genus *Plesiomonas*, including *P. shigelloides* , is higher, potentially causing diseases in captive YFP, a potential pathogen for humans and animals (Janda et al. 2016). To mitigate these risks, strict biosecurity measures including routine health screenings, dietary monitoring, and environmental disinfection were enforced throughout the study to minimize pathogen exposure and ensure the well‐being of the YFPs in captivity. *Clostridiaceae* in the small intestine are crucial for carbohydrate metabolism and may enhance nutrient acquisition in humans (Zoetendal et al. 2012) and were higher during the NB season. *Cetobacterium* increases in the B season compared to the NB season. Research suggests that *Cetobacterium* have probiotic properties and contribute to the host's metabolism (Wang et al. 2021; Colorado Gómez et al. 2023; Qi et al. 2023). In the intestinal microbiota of captive common bottlenose dolphins, Suzuki et al. (2022) discovered *Cetobacterium ceti* , which revealed vitamin B12 production, which is essential to supply dolphins with myoglobin and hemoglobin. LEfSe analysis revealed that *Staphylococcus* increased significantly during the breeding season while *Terrisporobacter* dominated in the nonbreeding season of male captive YFPs. The elevated *Staphylococcus* abundance during reproductive periods may relate to physiological changes in the urogenital tract, though this genus has been associated with deteriorated sperm quality in other mammals (Ďuračka et al. 2021), potentially indicating a trade‐off between reproductive activity and microbial impacts on fertility. In contrast, the NB season's predominance of *Terrisporobacter* likely supports host health maintenance, as this genus produces beneficial SCFAs through carbohydrate metabolism that enhance gut homeostasis and energy regulation (Wang et al. 2022). These seasonal microbial shifts suggest an adaptive interplay between reproduction and microbiota dynamics, where *Staphylococcus* may transiently affect reproductive output while *Terrisporobacter* promotes metabolic recovery during the NB season.

The present study compared the metabolites between the B and NB seasons using untargeted metabolomics, demonstrating PCA effectively differentiated metabolite spectra between the breeding seasons. Bile acid, a gut metabolite, plays a crucial role in lipid metabolism and fat digestion (Ahmad and Haeusler 2019; Yu et al. 2019). A bile acid derivative, 6,7‐diketolithocholic acid, was upregulated in the B season and was previously reported to be increased in obese pigs as compared to the lean pigs (Hu et al. 2024). Bufalin, a lipid‐like metabolite, is upregulated in the B season and has been shown to be effective in inhibiting various cancer types, including gastric, bladder, ovarian, liver, and breast cancer (Soumoy et al. 2022). It also stimulates cardiac contraction, regulates blood pressure, and reduces inflammation (Zheng et al. 2012; Wen et al. 2014). Dimerum acid, an organic acid, has been found to inhibit bile flow and biliary excretion, potentially contributing to obesity and bile GSH depletion‐related retro‐hepatic diseases (Yano et al. 2010), and was upregulated in the B season in the captive YFP. Thiomorpholine 3‐carboxylate, an organic acid derivative metabolite, is upregulated in the B season. Thiomorpholine 30‐carboxylate's free content is controlled by intracellular thiomorpholine–carboxylate dehydrogenases, which produce NADH and NADPH in response to energy metabolism changes (Nielsen et al. 2016). 4‐Oxo‐retinic acid is an active geometric isomer of retinoic acid (RA), which is affected by CYP26A1, CYP26B1, and CYP26C1, three types of cytochromes P450 (CYP26) enzymes. These enzymes play an important role in physiological processes, including energy metabolism, by affecting the metabolic activity of RA in cells and regulating gene expression (Berry et al. 2014; Zhang et al. 2018), which was upregulated in the B season, as compared to the NB season. An organoheterocyclic molecule known as mesobilirubinogen, which is proinflammatory and was downregulated in NB, may be crucial to the health of captive YFP during the NB season. Pimozide, a benzenoid class metabolite that inhibits cancer cell proliferation by reducing STAT (signal transducer and activator of transcription) activity (Hou et al. 2020), was downregulated in the NB season compared to the B season, potentially reflecting reduced metabolic demands for cell growth regulation outside the reproductive period. Similarly, lisinopril, an organic acid derivative with anti‐inflammatory effects via TNF‐α suppression (Morsy 2011), was also downregulated in the NB season, suggesting diminished need for inflammatory control during this phase of lowered physiological activity in captive YFP. Lisinopril, an organic acid derivative, has been found to have anti‐inflammatory effects by suppressing proinflammatory cytokines and was downregulated in the NB seasonal group. Leptomycin B, a metabolite from *Streptomyces* sp. strain ATS1287, is known for its antifungal properties (Shao et al. 2011) and was downregulated during the NB season, which may indicate its potential antifungal role in the captive YFP.

The DMs significantly altered the KEGG pathways in male captive YFP during the B and NB seasons. The amino acids and organic acids and derivatives (l‐tyrosine, l‐lysine, and n6‐acetyle‐l‐lysine) were downregulated during the NB seasons, affecting protein digestion and lysine degradation. L‐tyrosine raises testosterone levels and improves the semen quality in bulls (El‐Amrawi et al. 1995); in cows, one or two doses of l‐tyrosine result in increased growth and yield (El‐Hamd and Sayah 2015). Amino acid, L‐lysine increases intestinal calcium absorption, anxiety reduction, and muscle recovery in exercise (Pena et al. 2017; Hayamizu et al. 2019). L‐lysine and n6‐acetyle‐l‐lysine are crucial for energy production, providing nitrogen for nonessential amino acid synthesis. Acetyl‐CoA, a lysine degradation product, contributes to energy generation through the carnitine shuttle system, citric acid cycle, fat metabolism, and immune response (Li et al. 2017). Organic acids and derivatives, such as 5‐l‐glutamyl‐taurine, are intermediates in taurine and hypotaurine metabolism, produced from taurine via gamma‐glutamyltranspeptidase (Xu et al. 2020). Upregulation of 5‐l‐glutamyl‐taurine during the B season significantly alters taurine and hypotaurine metabolism, which was previously reported to generate energy for biological processes (Meng et al. 2023). The NB season exhibits a substantial rise in the biosynthesis of phenylalanine, tyrosine, and tryptophan, which are crucial for regulating inflammation, immune response, and oxidative stress (Anesi et al. 2019; Liu et al. 2019). L‐phenylalanine, an unstable amino acid, is converted to l‐tyrosine by phenylalanine hydroxylase (PAH), which can affect symbiotic bacteria and potentially alter energy metabolism, inflammation, intestinal permeability, and systemic immunity (Ashe et al. 2019; Dodd et al. 2017). In the B season, tyramine glucuronide and 15‐hydroxynorandrostene‐3,17‐dione glucuronide were upregulated, altering the bile secretion pathway. This suggests higher feed utilization efficiency in male captive YFP, as reported in commercial pigs (Wang et al. 2019) and male geese (Yang et al. 2024).

Researchers have reported a strong association between gut bacteria and metabolites, with gut microbiota affecting multiple metabolic pathways (Zhang et al. 2022). Seasonal breeding changes in fecal microbiota abundance led to changes in the metabolome. The study found a strong association between cyclohexane and *Clostridium_sensu_stricto_1*, which aligns with Shah, Tang, He, Hao, Ahmad, et al. (2024) findings, indicating that they could be important for the well‐being of the male captive YFP. L‐proline is a proteinogenic amino acid that plays a central role in protein synthesis (Moradi et al. 2022). Cyclohexane has adverse effects on the hippocampus of adult mice, causing excitotoxicity, motor impairment, and astrogliosis (Campos‐Ordonez et al. 2019). *Staphylococcus* genus species significantly contribute to deteriorated sperm quality and serve as reservoirs of antimicrobial‐resistant genes, posing a threat to animal and human health (Tvrdá et al. 2018; Ďuračka et al. 2021; Rossi et al. 2020). *Macrococcus*, a gram‐positive coccoid bacterium, is closely related to *Staphylococcus* but has its own genus since 1998 (Cotting et al. 2017). Current information on *Macrococcus* distribution is limited. *Staphylococcus* and *Macrococcus* were positively correlated with secalciferol, which may play a role in the physiological changes of male captive YFP. The study found that *Cardiobacteriaceae*, which are associated with bacteremia and wound infections (Das et al. 1997), were positively correlated with o‐acetylcarnitine metabolite. These bacteria were found in dolphin blowholes, tongues, and oral cavities (Godoy‐Vitorino et al. 2017), but healthy bottlenose dolphins also showed 51 OTUs belonging to the *Cardiobacteriaceae* family in their oral cavity (Bik et al. 2016). *Aeromonas*, *Ornithinibacter*, *Peptostreptococcaceae*, and *Trichococcus* are associated with deforolimus, taurohyocholate, l‐glutamic acid, and ergocornine. *Aeromonas* cause various human infectious diseases (Pessoa et al. 2019), while the *Peptostreptococcaceae* family are common gastrointestinal system residents. The *Peptostreptococcaceae* family is abundant in high‐fertility groups compared to low‐fertility in semen stallions (Malaluang et al. 2024).

Currently, few captive animals are trained for fecal sample collection, limiting our sample size. Future studies should include more animals to validate these results and specifically examine key gut microbes and its metabolites that may affect seasonal breeding behavior.

## 5 Conclusions

The study used the 16S rRNA and UHPLC–MS/MS techniques to investigate seasonal differences in the fecal microbiota and its metabolites of male captive YFP. Firmicutes are rich in the NB season, while Actinobacteria, Proteobacteria, and Fusobacteriota increase during the B season. *Clostridium_sensu_stricto_13*, *Paeniclostridium*, and *Mycobacterium* increase during the NB season, while *Romboutsia*, *Plesiomonas*, and *Cetobacterium* increase during the B season. The fecal metabolome undergoes significant changes during the B and NB seasons, altering metabolic pathways like protein digestion, phenylalanine metabolism, lysine degradation, taurine and hypotaurine metabolism, bile secretion, tyrosine metabolism, and tryptophan biosynthesis. The current study investigated potential regulatory mechanisms and examined the relationship between fecal microbiota and its metabolites and seasonal breeding. This research offers a new theoretical basis for YFP captive breeding and aids researchers in understanding seasonal changes in YFP metabolism. Future research should clarify the composition and function of fecal microbes and metabolites in captive YFP and other cetaceans and explore their role in seasonal breeding animals.

## Author Contributions

**Syed Ata Ur Rahman Shah:** conceptualization (equal), data curation (equal), formal analysis (equal), investigation (equal), methodology (equal), software (equal), validation (equal), visualization (equal), writing – original draft (equal). **Bin Tang:** conceptualization (equal), data curation (equal), formal analysis (equal), methodology (equal), writing – review and editing (equal). **Dekui He:** funding acquisition (equal), project administration (equal), supervision (equal), writing – review and editing (equal). **Maaz Ahmad:** methodology (equal). **Ghulam Nabi:** methodology (equal), writing – review and editing (equal). **Chaoqun Wang:** methodology (equal), resources (equal). **Zhangbing Kou:** methodology (equal), resources (equal). **Kexiong Wang:** funding acquisition (equal), project administration (equal), supervision (equal), writing – review and editing (equal). **Yujiang Hao:** conceptualization (equal), funding acquisition (equal), project administration (equal), supervision (equal), writing – review and editing (equal).

## Conflicts of Interest

The authors declare no conflicts of interest.

## Supporting information


Data S1

## Data Availability

The data supporting the findings of this study are included within the article and its Data S1. The raw data supporting the findings of this study have been deposited in the National Center for Biotechnology Information (NCBI) under the BioProject accession number PRJNA1242582.
