# Supplementary material for: Seasonal Breeding Alters Fecal Microbiota and Metabolome in the Male Captive Yangtze Finless Porpoise (Neophocaena asiaeorientalis asiaeorientalis)
Source: Ecol Evol. 2025 Jun 22;15(6):e71611. doi: 10.1002/ece3.71611 (PMC12182982; doi:10.1002/ece3.71611)
Supplement: Supplementary file 1 — Data S1 [file ECE3-15-e71611-s001.zip › ece371611-sup-0001-Supinfo.docx]

**Figure S1: (a)** A microbiota rarefaction curve derived from B and NB season samples using the Shannon index. **(b)** A Venn diagram illustrating the overlapping numbers of OTUs between the captive YFP’s B and NB groups.

**Figure S2:** Alpha diversity of the captive YFP’s gut microbiota. (a) OTU-level ACE index. (b) OTU-level Chao index. (c) OTU-level Shannon index. (d) OTU-level Simpson index. Significant differences between the B and NB groups in the male captive YFP were tested by Student’s t-test.

**Table S2:** The gut microbiomes alpha-diversity in the captive YFP’s B and NB groups.

|  | **B Season** | **NB Season** | ***p*-value** |
| --- | --- | --- | --- |
| **Ace** | 246.97 | 188.5 | 0.5489 |
| **Chao** | 220.21 | 174.14 | 0.5647 |
| **Coverage** | 0.9989 | 0.99915 | 0.5489 |
| **Shannon** | 1.4087 | 1.1133 | 0.5489 |
| **Simpson** | 0.40344 | 0.49838 | 0.5489 |
| **Sobs** | 176.9 | 145 | 0.5701 |
